# In-Hospital and One-Year Mortality and Their Predictors in Patients Hospitalized for First-Ever Chronic Obstructive Pulmonary Disease Exacerbations: A Nationwide Population-Based Study

**DOI:** 10.1371/journal.pone.0114866

**Published:** 2014-12-09

**Authors:** Te-Wei Ho, Yi-Ju Tsai, Sheng-Yuan Ruan, Chun-Ta Huang, Feipei Lai, Chong-Jen Yu, The HINT Study Group

**Affiliations:** 1 Graduate Institute of Biomedical Electronics and Bioinformatics, National Taiwan University, Taipei, Taiwan; 2 School of Medicine, College of Medicine, Fu-Jen Catholic University, New Taipei, Taiwan; 3 Department of Internal Medicine, National Taiwan University Hospital, Taipei, Taiwan; 4 Department of Traumatology, National Taiwan University Hospital, Taipei, Taiwan; 5 Graduate Institute of Clinical Medicine, National Taiwan University, Taipei, Taiwan; 6 Department of Computer Science and Information Engineering, National Taiwan University, Taipei, Taiwan; 7 Department of Electrical Engineering, National Taiwan University, Taipei, Taiwan; 8 Hospitalist in National Taiwan University Hospital, Taipei, Taiwan; Pulmonary Medicine, China

## Abstract

**Introduction:**

Natural history of chronic obstructive pulmonary disease (COPD) is punctuated by exacerbations; however, little is known about prognosis of the first-ever COPD exacerbation and variables predicting its outcomes.

**Materials and Methods:**

A population-based cohort study among COPD patients with their first-ever exacerbations requiring hospitalizations was conducted. Main outcomes were in-hospital mortality and one-year mortality after discharge. Demographics, comorbidities, medications and in-hospital events were obtained to explore outcome predictors.

**Results:**

The cohort comprised 4204 hospitalized COPD patients, of whom 175 (4%) died during the hospitalization. In-hospital mortality was related to higher age (odds ratio [OR]: 1.05 per year; 95% confidence interval [CI]: 1.03–1.06) and Charlson comorbidity index score (OR: 1.08 per point; 95% CI: 1.01–1.15); angiotensin II receptor blockers (OR: 0.61; 95% CI: 0.38–0.98) and β blockers (OR: 0.63; 95% CI: 0.41–0.95) conferred a survival benefit. At one year after discharge, 22% (871/4029) of hospital survivors were dead. On multivariate Cox regression analysis, age and Charlson comorbidity index remained independent predictors of one-year mortality. Longer hospital stay (hazard ratio [HR] 1.01 per day; 95% CI: 1.01–1.01) and ICU admission (HR: 1.33; 95% CI: 1.03–1.73) during the hospitalization were associated with higher mortality risks. Prescription of β blockers (HR: 0.79; 95% CI: 0.67–0.93) and statins (HR: 0.66; 95% CI: 0.47–0.91) on hospital discharge were protective against one-year mortality.

**Conclusions:**

Even the first-ever severe COPD exacerbation signifies poor prognosis in COPD patients. Comorbidities play a crucial role in determining outcomes and should be carefully assessed. Angiotensin II receptor blockers, β blockers and statins may, in theory, have dual cardiopulmonary protective properties and probably alter prognosis of COPD patients. Nevertheless, the limitations inherent to a claims database study, such as the diagnostic accuracy of COPD and its exacerbation, should be born in mind.

## Introduction

Chronic obstructive pulmonary disease (COPD), according to the definition by the Global initiative for chronic Obstructive Lung Disease (GOLD), is a common preventable and treatable disease, characterized by persistent airflow limitation that is usually progressive and associated with chronic airway and lung inflammatory responses. [1] This disease is one of the leading cause of morbidity and mortality worldwide and poses a huge burden on economy and society. [1],[2] An exacerbation of COPD is characterized by acute worsening of respiratory symptoms that is beyond normal daily variations and leads to alterations of drug therapy. [1] The natural history of COPD is punctuated by exacerbations that account for the largest part of the total COPD burden on the healthcare system. [1] Moreover, exacerbations result in impaired physical activity, poorer life quality and increased death risk of COPD patients.[3]–[5] Over the past decades, a number of studies have put much effort into studying outcomes and their predictors of COPD exacerbations; [6] however, few of them specifically focus on first episodes of COPD exacerbations. [7] Knowledge about prognosis of the first-ever COPD exacerbation and factors that predict poor outcomes is of paramount importance because this enables physicians to educate patients about harms of a COPD exacerbation and to reinforce their compliance of treatment programs before they experience it themselves. Such information is also vital to help make crucial management decisions such as intensity of follow-up visits and decisions to escalate or withdraw treatment.

Therefore, the aim of the present study is to describe the in-hospital and one-year outcomes and to investigate their predictors in patients with the first hospitalization for COPD exacerbations using a large population-based database.

## Materials and Methods

### Study Design and Data Source

We conducted a retrospective population-based cohort study using the Longitudinal Health Insurance Database (LHID) from 2000 to 2008. Taiwan launched a mandatory National Health Insurance (NHI) program in 1995, which founded on the principle that every citizen should have equal access to healthcare. At the end of 2011, up to 99.9% of the 23 million people were enrolled in the NHI program. [8] For the purpose of research and policy assessment, the National Health Insurance Administration collaborated with the National Health Research Institutes to construct the National Health Insurance Research Database and released original claims data since 2000. [9] The LHID consisted of one million subjects who were randomly selected from the entire NHI beneficiaries, with the details of each visit record, including ambulatory care expenditures and orders, and inpatient expenditures and orders, and registry for beneficiaries. The LHID was considered to have representative power of the national population. [9] To protect privacy, the sensitive information, such as identification of subjects, medical institutions and medical staff, was encrypted. The research ethics committee of the National Taiwan University Hospital waived the need for review board approval and written informed consent.

### Study Population

The study cohort consisted of all patients who had been hospitalized for COPD exacerbations between January 2005 and December 2007, and the patients were followed up till the end of 2008. Although only the International Classification of Diseases, Ninth Revision, Clinical Modification (ICD-9-CM) code of 491.21 denotes pure COPD exacerbations, we defined hospitalizations for COPD exacerbations as patients admitted with a primary diagnosis of COPD (ICD-9-CM codes of 490, 491, 492, 496) or those with a primary diagnosis of pneumonia (ICD-9-CM codes of 480–486) and a secondary diagnosis of COPD in this study.[10]–[13] We included the pneumonia codes because it is often difficult to decide if COPD exacerbations are accompanied with pneumonia or not, and co-existence of COPD exacerbations and pneumonia is common. [10], [12], [14] The study population also had to have at least two outpatient visits for COPD and to be dispensed at least two COPD-related medications within one year. During the study period, the first admission for COPD exacerbations was defined as the index hospitalization. Patients were excluded if they were aged <40 years at the index hospitalization or had a history of admission for COPD exacerbations after year 2000 and before cohort entry.

### Data Collection and Outcomes

The outcomes of interest were in-hospital mortality and one-year mortality after discharge. The study cohort was followed from the date of the index hospitalization to one year after discharge or until death, whichever came first. The demographics, comorbid diseases, concomitant medications and COPD medications were also identified from the LHID. The COPD-related medications, including short-acting and long-acting β_2_ agonists, anticholinergics, inhaled corticosteroids and theophyllines, were measured in the 6 months before the index hospitalization. Comorbidities as detailed in S1 Table were noted if they were present prior to the index hospitalization. The Charlson comorbidity index was calculated as previously described. [15] We also collected information about the index hospitalization, including the duration of hospital stay, frequency and length of intensive care unit (ICU) admission, acute cardiovascular events, use of non-invasive ventilatory support, and use of mechanical ventilation and ventilator days. The cardiovascular events of interests included acute myocardial infarction (ICD-9-CM code of 410), unstable angina (ICD-9-CM code of 411.1), acute heart failure (ICD-9-CM codes of 428.1, 428.21, 428.23, 428.31, 428.33, 428.41, 428.43), transient ischemic attack (ICD-9-CM codes of 435, 437.1), and ischemic stroke (ICD-9-CM codes of 433.x1, 434.x1, 436). Since spirometry results were not available in the LHID, proxy indicators of COPD severity, including the number of types of COPD medications prescribed (≤2 vs. >2) and number of COPD-related emergency visits (≤2 vs. >2) were measured during the 6-month period before the index hospitalization. In addition, medications dispensed to the patients on hospital discharge were recorded and patient compliance with discharge medications was determined by estimating the medication possession ratio, which was calculated as the sum of the days supply for all claims during a defined period of time divided by the number of days elapsed during the period. [16] Medication possession ratios of ≧0.8, ≧0.5 to <0.8 and <0.5 indicated good, moderate and poor compliance, respectively. [17].

### Statistical Analysis

Qualitative variables were expressed in percentages, and quantitative variables were summarized as mean ± standard deviation or as median and interquartile range as appropriate. To compare continuous variables, the Student’s t-test or Mann-Whitney U test were used. Categorical data were analyzed by the chi-square test. Independent predictors of in-hospital mortality were identified by use of multivariate logistic regression analysis. The multivariate relationship between survival time and covariates was determined with the Cox regression analysis model. Survival time was defined as the interval between hospital discharge and the date of death, or censored at one year if they remained alive at that time. Potential covariates were entered into the multivariate models if they were statistically significant in the univariate analysis. The survival curve was plotted using the Kaplan-Meier method. Data analyses were performed using SPSS software (Version 15.0, SPSS Inc., Chicago, IL, USA). A 2-sided P value of <0.05 was considered statistically significant.

## Results

### Characteristics of Patients and Index Hospitalizations

**Figure 1:**
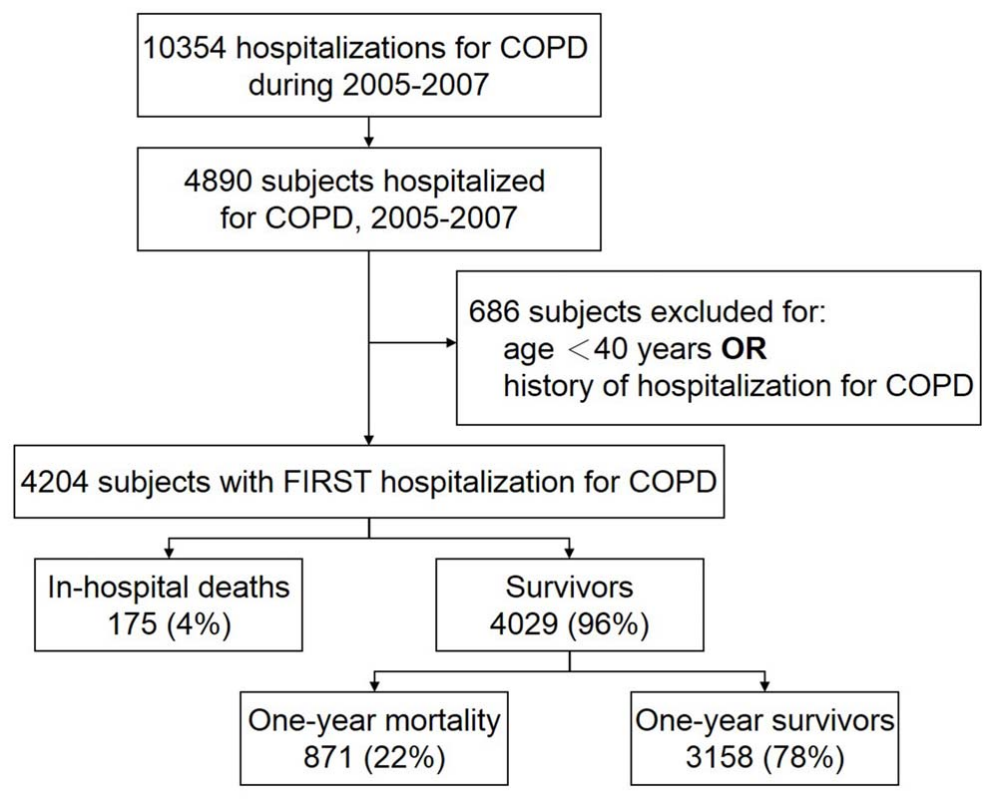
Study flow diagram and main outcomes. COPD, chronic obstructive pulmonary disease.

During 2005–2007, a total of 4204 study patients, 155 (3.7%), 2570 (61.1%), 36 (0.9%) and 1443 (34.3%) with ICD-9-CM codes of 490, 491, 492 and 496, respectively, were included in the study (Fig. 1). The baseline characteristics of the patients are reported in Table 1. On cohort entry, the mean age of the entire population was 75 years and 73% of them were male. The majority of the patients had comorbidities and the average score on the Charlson comorbidity index was 3.6. The most commonly observed comorbidities were hypertension (65%), coronary artery disease (37%) and stroke (32%). About three-fourths of the patients were placed on ≤2 COPD medications and the number of emergency visits for COPD was ≤2 in 80% of patients. The length of stay in hospitals was 12±20 days (Table 2). Ten percent (429/4204) of patients had been admitted to the ICU with an average stay of 8 days. Mechanical ventilation was required in 350 (8.3%) patients and the median duration of ventilatory support was 7 days. One hundred sixty-five (3.9%) patients were placed on non-invasive ventilation.

**Table 1:** Baseline characteristics of the study population with chronic obstructive pulmonary disease with regard to the in-hospital outcome.

		In-hospital outcome		
	Total	Survivor	Nonsurvivor	
Characteristics	(n = 4204)	(n = 4029)	(n = 175)	P value
Age, years	75±11	75±11	80±9	<0.001
Male sex	3066 (73)	2947 (73)	119 (68)	0.134
Charlson comorbidity index	3.6±2.7	3.6±2.7	4.4±2.9	<0.001
Comorbidities				
Coronary artery disease	1574 (37)	1514 (38)	60 (35)	0.402
Depressive disorder	380 (9.0)	367 (9.1)	13 (7.4)	0.458
Diabetes mellitus	1163 (28)	1119 (28)	44 (25)	0.467
End-stage renal disease	261 (6.2)	247 (6.1)	14 (8.0)	0.308
Heart failure	921 (22)	870 (22)	51 (29)	0.016
Hyperlipidemia	823 (20)	799 (20)	24 (14)	0.049
Hypertension	2719 (65)	2605 (65)	114 (66)	0.833
Liver cirrhosis	101 (2.4)	99 (2.5)	2 (1.1)	0.269
Malignancy	440 (10)	412 (10)	28 (16)	0.014
Stroke	1331 (32)	1262 (31)	69 (40)	0.021
Co-medications				
ACEI	891 (21)	849 (21)	42 (24)	0.201
ARB	759 (18)	738 (18)	21 (12)	0.018
Antiplatelet	1579 (37)	1516 (37)	63 (36)	0.363
β blocker	1005 (23)	977 (24)	28 (16)	0.006
Statin	288 (6.9)	284 (7.0)	4 (2.3)	0.006
COPD medications				
SABA	1767 (42)	1690 (42)	77 (44)	0.590
LABA	508 (12)	487 (12)	21 (12)	0.972
Anticholinergic	1164 (28)	1111 (28)	53 (30)	0.433
ICS	571 (14)	548 (14)	23 (13)	0.862
Theophylline	2403 (57)	2313 (57)	90 (51)	0.118
COPD severity proxy indicators				
COPD medications				
≤2	3093 (74)	2965 (74)	128 (73)	0.895
>2	1111 (26)	1064 (26)	47 (27)	
COPD-related emergency visits				
≤2	3363 (80)	3221 (80)	141 (81)	0.698
>2	841 (20)	808 (20)	33 (19)	

Data are presented as mean ± standard deviation or number (%).

ACEI, angiotensin converting enzyme inhibitor; ARB, angiotensin II receptor blocker; COPD, chronic obstructive pulmonary disease; ICS, inhaled corticosteroid; LABA, long-acting β_2_ agonist; SABA, short-acting β_2_ agonist.

**Table 2:** Major events during the index hospitalization regarding the in-hospital outcome.

		In-hospital outcome		
	Total	Survivor	Nonsurvivor	
Events	(n = 4204)	(n = 4029)	(n = 175)	P value
Length of hospital stay, days	12±20	11±15	31±63	<0.001
ICU admission	429 (10)	352 (8.7)	77 (44)	<0.001
Length of ICU stay, days	8±9	7±8	11±12	0.004
MV	350 (8.3)	246 (6.1)	104 (59)	<0.001
Duration of MV, days	7 (2–19)	6 (3–16)	10 (1–28)	0.552
Non-invasive ventilation	165 (3.9)	131 (3.3)	34 (19)	<0.001
Cardiovascular events	144 (3.4)	132 (3.3)	12 (6.9)	0.011

Data are presented as mean ± standard deviation, number (%) or median (interquartile range).

ICU, intensive care unit; MV, mechanical ventilation; NIV, non-invasive ventilation.

### In-hospital Outcome

During the index hospitalization, 175 (4%) of patients died (Table 1). The nonsurvivors were older and had a higher Charlson comorbidity index score than survivors. Comorbidities, including heart failure, malignancy and stroke, were more commonly seen in nonsurvivors; instead, a higher proportion of survivors had hyperlipidemia. Angiotensin II receptor blockers, β blockers and statins were more commonly prescribed in patients surviving the index hospitalization. As expected, nonsurvivors had a longer hospital length of stay and were more likely to require ICU admission and ventilatory support (Table 2). In addition, more cardiovascular events occurred during the hospital stay among the nonsurvivors. Multivariate logistic regression analysis showed that a higher age and Charlson comorbidity index score independently predicted in-hospital mortality (Table 3). In addition, the use of angiotensin II receptor blockers or β blockers was associated with lower in-hospital mortality.

**Table 3:** Logistic regression analysis of variables predictive of in-hospital mortality in patients admitted with chronic obstructive pulmonary disease.

Variables	Odds ratio	95% CI	P value
Age, per year	1.05	1.03–1.06	<0.001
CCI, per point	1.08	1.01–1.15	0.016
Heart failure	1.34	0.94–1.92	0.104
Hyperlipidemia	0.82	0.52–1.30	0.397
Malignancy	1.39	0.89–2.19	0.153
Stroke	1.19	0.85–1.66	0.320
Use of ARB	0.61	0.38–0.98	0.040
Use of β blocker	0.63	0.41–0.95	0.028
Use of statin	0.40	0.14–1.14	0.087

ARB, angiotensin II receptor blocker; CCI, Charlson comorbidity index; CI, confidence interval.

### One-year Outcome

**Figure 2:**
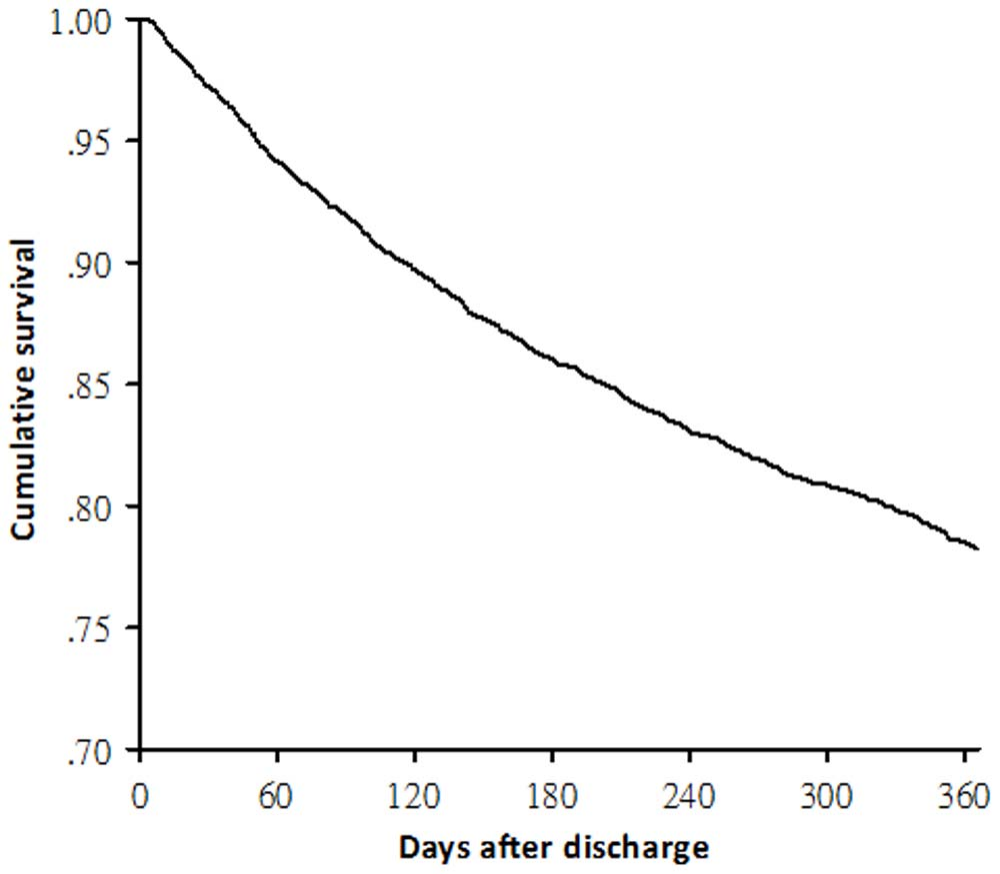
Survival curve for patients surviving first episodes of chronic obstructive pulmonary disease exacerbations.

A total of 3158 (78%) patients were alive at one year after discharge, as shown in Fig. 2. The demographics and clinical parameters were compared between patient groups categorized by one-year outcome (Table 4). On multivariate Cox regression analysis, age, Charlson comorbidity index, concomitant liver cirrhosis and malignancy, and length of stay and ICU admission during the index hospitalization were significant independent predictors of one-year mortality (Table 5). The prescription of β blockers and statins on hospital discharge were protective against mortality in the one-year follow-up. The proportion of patients with different medication compliance was displayed in S2 Table. When only patients with good or moderate compliance were regarded as drug users, similar protective effects of β blockers and statins on one-year mortality were observed (S3 Table).

**Table 4:** Characteristics of patients survived to hospital discharge with respect to the one-year outcome.

	One-year outcome		
	Survivor	Nonsurvivor	
Characteristics	(n = 3158)	(n = 871)	P value
Age, years	74±11	79±9	<0.001
Male sex	2289 (73)	658 (76)	0.071
Charlson comorbidity index	3.4±2.6	4.2±2.9	<0.001
Comorbidities			
Coronary artery disease	1180 (37)	334 (38)	0.619
Depressive disorder	286 (9.1)	81 (9.3)	0.835
Diabetes mellitus	852 (27)	267 (31)	0.034
End-stage renal disease	187 (5.9)	60 (6.9)	0.297
Heart failure	658 (21)	212 (24)	0.028
Hyperlipidemia	674 (21)	125 (14)	<0.001
Hypertension	2005 (64)	600 (69)	0.004
Liver cirrhosis	67 (2.1)	32 (3.7)	0.009
Malignancy	273 (8.7)	139 (16)	<0.001
Stroke	925 (29)	337 (39)	<0.001
Medications at discharge			
ACEI	740 (23)	215 (25)	0.442
ARB	672 (21)	179 (21)	0.641
Antiplatelet	1328 (42)	377 (43)	0.515
β blocker	849 (27)	192 (22)	0.004
Statin	280 (8.9)	42 (4.8)	<0.001
SABA	1546 (49)	417 (48)	0.573
LABA	538 (17)	101 (12)	<0.001
Anticholinergic	1063 (34)	309 (36)	0.317
ICS	590 (19)	104 (12)	<0.001
Theophylline	2152 (68)	571 (66)	0.149
Hospital events			
Length of hospital stay, days	10±11	15±25	<0.001
ICU admission	237 (7.5)	115 (13)	<0.001
Length of ICU stay, days	20±25	24±23	0.131
MV	159 (5.0)	87 (10)	<0.001
Duration of MV, days	6 (2–15)	9 (3–20)	0.139
Non-invasive ventilation	94 (3.0)	37 (4.2)	0.061
Cardiovascular events	94 (3.0)	38 (4.4)	0.042
COPD severity proxy indicators			
COPD medications			
≤2	2316 (73)	649 (75)	0.486
>2	842 (27)	222 (26)	
COPD-related emergency visits			
≤2	2523 (80)	698 (80)	0.873
>2	635 (20)	173 (20)	

Data are presented as mean ± standard deviation, number (%) or median (interquartile range).

ACEI, angiotensin converting enzyme inhibitor; ARB, angiotensin II receptor blocker; COPD, chronic obstructive pulmonary disease; ICS, inhaled corticosteroid; ICU, intensive care unit; LABA, long-acting β_2_ agonist; MV, mechanical ventilation; SABA, short-acting β_2_ agonist.

**Table 5:** Cox regression analysis of factors associated with one-year mortality in patients surviving exacerbations of chronic obstructive pulmonary disease.

Variables	Hazard ratio	95% CI	P value
Age, per year	1.04	1.03–1.05	<0.001
CCI, per point	1.06	1.03–1.09	<0.001
Comorbidities			
Liver cirrhosis	1.64	1.14–2.35	0.007
Hyperlipidemia	0.75	0.61–0.92	0.006
Malignancy	1.56	1.28–1.90	<0.001
Medications at discharge			
β blocker	0.79	0.67–0.93	0.005
Statin	0.66	0.47–0.91	0.013
In-hospital events			
Length of hospital stay, per day	1.01	1.01–1.01	<0.001
ICU admission	1.33	1.03–1.73	0.030

CCI, Charlson comorbidity index; CI, confidence interval; ICU, intensive care unit.

## Discussion

In this population-based cohort study, we provide information about the first-ever hospitalizations for a COPD exacerbation. The main findings are summarized as follows: (i) During the index hospital stay, 10% of the patients required ICU admission, 8.3% had been placed on mechanical ventilation and the in-hospital mortality rate was 4.2%. (ii) The age and comorbidity index were independent in-hospital mortality predictors; the use of angiotensin II receptor blockers and β blockers was found to be associated with a reduction in the risk of in-hospital mortality. (iii) Among patients surviving the index hospitalization, 22% of them died within one year of discharge. (iv) Increased age, a higher comorbidity index, the presence of chronic comorbid conditions, such as liver cirrhosis and malignancy, and a longer hospital stay and ICU admission during the index hospitalization were associated with a higher mortality risk at one year. Statin or β blocker use may be beneficial in COPD patients with respect to the one-year outcome following discharge.

Although COPD exacerbations have been widely studied, the exacerbation under study is seldom the first in the patient’s disease course. Recently, a study on first-ever hospitalizations for COPD demonstrated the risk of subsequent severe exacerbations and long-term mortality of these patients. [7] Our study further provides insight into short-term and long-term mortality risk factors for a first-ever hospitalization for COPD exacerbations and describes major events, namely in-hospital mortality, and need for ICU care and ventilatory support, during the index hospital stay. Both studies had similar patient age and one-year mortality rates. In addition, both found that higher age and comorbidity indexes were significant mortality predictors. Thus, the two studies collaborated to produce a more comprehensive picture of a first-ever severe exacerbation for physicians taking care of COPD patients.

Our study shows the high prevalence of comorbidities in patients hospitalized for COPD exacerbations and their importance in relation to in-hospital and one-year prognosis in this population. In stable COPD patients, the comorbidity severity, measured by the Charlson comorbidity index, is a well-established mortality predictor. [18], [19] However, the evidence is less consistent for hospitalized COPD exacerbations. A number of studies showed no independent association between the comorbidity burden and in-hospital [20] or longer-term mortality, [21], [22] although various comorbidity indexes have been shown to be independently predictive of in-hospital death[23]–[25] and post-discharge mortality. [26] The discrepancies may be explained by differences in the study design, source population and scoring system for comorbidities. In reality, over the past decade, the GOLD has put more and more emphasis on recognition and management of comorbid illnesses in COPD because of their potential impact on patient prognosis. [1] Our findings consolidate the idea that an individual patient’s health status plays a significant role in determining mortality risk in COPD patients.

COPD is commonly associated with cardiovascular diseases, such as coronary artery disease, heart failure, hypertension and stroke, because of shared risk factors or a causal relationship. [1], [27] Angiotensin II receptor blockers, β blockers and statins are commonly used therapeutic drug classes in the management of cardiovascular patients and many trials have demonstrated their protective effects on cardiovascular outcomes.[28]–[30] We found here that these medications were also associated with favorable outcomes in patients with severe COPD exacerbations. Statins, as a class of lipid-lowering drugs, have an additional immunomodulatory effect that may reduce neutrophil infiltration, cytokine production and matrix remodeling in COPD; [31] thus, it is biologically plausible that statin use is associated with a decreased risk of COPD exacerbations and mortality. [14], [32] Although a recent large-scale randomized controlled trial disapproved this concept, [33] it is still urged to dispense a statin for COPD patients with other indications for it. The use of β blockers has been traditionally considered a contraindication for COPD patients; [34] however, several studies have advocated that it is safe and even advantageous to initiate or continue β blocker therapy in COPD patients with or without an exacerbation. [20], [35] Apart from cardiovascular protection of β blockers, they may theoretically exert beneficial effects in COPD patients by counteracting sympathetic tone or ameliorating ischemic burden. [36] Furthermore, as suggested in asthma, chronic dosing of β blockers in COPD patients could confer certain bronchoprotective effects, such as reduced inflammation, mucous metaplasia and expression of various spasmogenic proteins, via the upregulation of β_2_ adrenoceptors. [37] The renin-angiotensin system is potentially implicated in the COPD pathogenesis through its involvement in the regulation of pro-inflammatory mediators in the lung. [38] Specifically, angiotensin II stimulates the release of interleukin-6, tumor necrosis factor-α and monocyte chemotactic protein-1, and has an immunomodulatory effect on T cell responses that mediate the lung injury in COPD. [38], [39] A recent study has shown that angiotensin II receptor blockers inhibited the cytokine response of type I alveolar cells to lung injury. [40] Our findings and clinical observations support the beneficial role of angiotensin II receptor blockers in COPD patients. [32], [41] Taken together, the favorable effects of these agents on COPD outcomes are worth particular attention. On one hand, they indicate the importance of comorbidity management in the care of COPD. On the other hand, these medications may, in theory, have direct pulmonary protective properties and alter the prognosis of COPD patients.

In line with prior studies, [20],[25],[42],[43] the present study shows that patients with a longer hospital stay and ICU admission had a worse in-hospital outcome during an admission for COPD exacerbations. We also demonstrate that the two variables were independent mortality predictors at one year following discharge. Both longer hospital length of stay and ICU admission reflect the severity of acute illnesses, that has a negative impact on in-hospital prognosis and, to a lesser degree, the outcome after discharge. [44] Thus, the findings in this study indicate that it may be helpful and important to commence post-discharge case management in patients experiencing ICU care or prolonged hospital stay during a COPD exacerbation in hope of improving long-term prognosis.

We found mortality in the first year after hospital discharge for COPD exacerbations to be 22%, a rate similar to that reported by another study. [7] However, our one-year mortality rate seemed not to be lower than those in other series that also included patients with prior severe COPD exacerbations. [18], [26] Indeed, our study population had older age, an independent predictor of post-discharge mortality, [44] compared to other study subjects, but our results re-emphasize the impact of a hospitalized COPD exacerbation even it is the first-ever one. In short, other studies [7], [45] and ours suggest that hospitalizations for a COPD exacerbation identify a COPD subpopulation with a poor outcome. Based on the present study, it is suggested that more intensive plans and follow-up may be required for these high-risk patients, particularly if they were older, comorbid or discharged after a COPD hospitalization involving long hospital stay or ICU treatment.

There were some limitations to this study. First, the accuracy of COPD diagnosis depends on proper ICD-9-CM coding. Although the lack of unique patient identifiers in the LHID prevented a validation study, the database has been widely used to study COPD. [14], [46] In addition, we did not only rely on ICD-9 codes to identify COPD patients, but the use of COPD medications and an age of 40 or more years were mandatory elements. Second, the information on the severity of symptoms and exercise intolerance, and the data of pulmonary function testing were not available in the LHID; however, the proxy indicators for COPD severity and use of concomitant medications were controlled in the analysis. Third, cultural differences may hinder the generalization of our results. For example, rather than use inhaled drugs, Chinese people prefer to take oral medications. Thus, compared to studies conducted in western countries, [47] inhaled corticosteroids were less commonly prescribed and theophyllines were more frequently dispensed to our patients. In addition to clinical variables, several metabolic, physiological and hemodynamic factors were also found to have effects on the in-hospital mortality, [24], [48] but these variables were not retrievable from the LHID. This is the inborn disadvantage of being a claims database study; however, this kind of study provides an unbiased population cohort for medical researches and offers a powerful means of generating evidence to devise healthcare strategies.

In conclusion, an exacerbation requiring hospitalization denotes a poor long-term outcome in COPD patients; even it is the first-ever one. The burden of comorbidities has a significant role in determining mortality risks, and should be carefully evaluated and managed. Angiotensin II receptor blockers, β blockers and statins may theoretically have dual cardiopulmonary protective effects and probably improve outcomes of a severe exacerbation in patients with COPD. Nevertheless, the limitations inherent to a claims database study, such as the diagnostic accuracy of COPD and its exacerbation, should be born in mind.

## Supporting Information

S1 Table **Definitions of comorbidities.**
(DOC)

S2 Table **Compliance categories for discharge medications.**
(DOC)

S3 Table **Cox regression analysis of factors associated with one-year mortality in patients surviving exacerbations of chronic obstructive pulmonary disease while only patients with good or moderate medication compliance were regarded as drug users.**
(DOC)
