# Supplementary material for: In-Hospital and One-Year Mortality and Their Predictors in Patients Hospitalized for First-Ever Chronic Obstructive Pulmonary Disease Exacerbations: A Nationwide Population-Based Study
Source: PLoS One. 2014 Dec 9;9(12):e114866. doi: 10.1371/journal.pone.0114866 (PMC4260959; doi:10.1371/journal.pone.0114866)
Supplement: S1 Table — Definitions of comorbidities. (DOC) [file pone.0114866.s001.doc]

Table S1. Definitions of comorbidities.

| Comorbidities | ICD-9-CM codes |
| --- | --- |
| Coronary artery disease | 410-414 |
| Depressive disorder | 296.2, 296.3, 298.0, 300.4, 301.12, 309.28, 311, V79.0 |
| Diabetes mellitus | 250 |
| End-stage renal disease | 403.01, 403.11, 403.91, 404.02, 404.03, 404.12, 404.13, 404.92, 404.93, 585 |
| Heart failure | 402.01, 402.11, 402.91, 404.01, 404.03, 404.11, 404.13, 404.91, 404.93, 428 |
| Hyperlipidemia | 272 |
| Hypertension | 401-405 |
| Liver cirrhosis | 571.2, 571.5, 571.6 |
| Malignancy | 140-208 |
| Stroke | 430-437 |

ICD-9-CM, International Classification of Diseases, Ninth Revision, Clinical Modification.
