# Supplementary material for: In-Hospital and One-Year Mortality and Their Predictors in Patients Hospitalized for First-Ever Chronic Obstructive Pulmonary Disease Exacerbations: A Nationwide Population-Based Study
Source: PLoS One. 2014 Dec 9;9(12):e114866. doi: 10.1371/journal.pone.0114866 (PMC4260959; doi:10.1371/journal.pone.0114866)
Supplement: S2 Table — Compliance categories for discharge medications. (DOC) [file pone.0114866.s002.doc]

Table S2. Compliance categories for discharge medications.

|  |  | Compliance |  |
| --- | --- | --- | --- |
| Discharge medications | Good | Moderate | Poor |
| ACEI | 381 (40) | 377 (39) | 197 (21) |
| ARB | 347 (41) | 340 (40) | 164 (19) |
| Antiplatelet | 668 (39) | 691 (41) | 346 (20) |
| β blocker | 425 (41) | 426 (41) | 190 (18) |
| Statin | 127 (39) | 123 (38) | 72 (22) |
| SABA | 646 (33) | 610 (31) | 707 (36) |
| LABA | 236 (37) | 238 (37) | 165 (26) |
| Anticholinergic | 453 (33) | 450 (33) | 469 (34) |
| ICS | 259 (37) | 233 (34) | 202 (29) |
| Theophylline | 1078 (40) | 1110 (41) | 535 (20) |

ACEI, angiotensin converting enzyme inhibitor; ARB, angiotensin II receptor blocker; ICS, inhaled corticosteroid; LABA, long-acting β_2_ agonist; SABA, short-acting β_2_ agonist.
