# Supplementary material for: In-Hospital and One-Year Mortality and Their Predictors in Patients Hospitalized for First-Ever Chronic Obstructive Pulmonary Disease Exacerbations: A Nationwide Population-Based Study
Source: PLoS One. 2014 Dec 9;9(12):e114866. doi: 10.1371/journal.pone.0114866 (PMC4260959; doi:10.1371/journal.pone.0114866)
Supplement: S3 Table — Cox regression analysis of factors associated with one-year mortality in patients surviving exacerbations of chronic obstructive pulmonary disease while only patients with good or moderate medication compliance were regarded as drug users. (DOC) [file pone.0114866.s003.doc]

Table S3. Cox regression analysis of factors associated with one-year mortality in patients surviving exacerbations of chronic obstructive pulmonary disease while only patients with good or moderate medication compliance were regarded as drug users.

| Variables | Hazard ratio | 95% CI | P value |
| --- | --- | --- | --- |
| Age, per year | 1.04 | 1.03-1.05 | <0.001 |
| CCI, per point | 1.06 | 1.03-1.10 | <0.001 |
| Comorbidities |  |  |  |
| Liver cirrhosis | 1.67 | 1.16-2.39 | 0.005 |
| Hyperlipidemia | 0.77 | 0.63-0.95 | 0.012 |
| Malignancy | 1.55 | 1.27-1.90 | <0.001 |
| Medications at discharge |  |  |  |
| β blocker | 0.62 | 0.51-0.75 | <0.001 |
| Statin | 0.50 | 0.33-0.76 | 0.001 |
| In-hospital events |  |  |  |
| Length of hospital stay, per day | 1.01 | 1.01-1.01 | <0.001 |
| ICU admission | 1.36 | 1.05-1.76 | 0.020 |

CCI, Charlson comorbidity index; CI, confidence interval; ICU, intensive care unit.
